# Investigating the purpose of an online discussion group for health professionals: a case example from forensic occupational therapy

**DOI:** 10.1186/1472-6963-13-253

**Published:** 2013-07-03

**Authors:** Crystal Dieleman, Edward AS Duncan

**Affiliations:** 1 School of Occupational Therapy, Dalhousie University, 5869 University Avenue, PO box 15000, Halifax NS B3H 4R2, Canada; 2 Nursing Midwifery and Allied Health Professions Research Unit, Iris Murdoch Building University of Stirling, Stirling FK9 4LA, UK

**Keywords:** Online discussion group, Forensic occupational therapy, Communication, Networking

## Abstract

**Background:**

Thousands of health-related online discussion groups are active world-wide however, very little is known about the purpose and usefulness of such groups. In 2003 an online discussion group called ‘forensic occupational therapy’ was established in the United Kingdom. This group was examined to gain an understanding of the purpose and use of online discussion groups for health professionals who may be practically and geographically isolated from others in similar areas of practice.

**Methods:**

Following a case study design, descriptive characteristics on members’ locations and number of posts were collected from the forensic occupational therapy online discussion group. Eight years of posts (2003–2011) were examined using a theoretical thematic analysis process to identify and describe the purposes for which members were using the group.

**Results:**

Members from 20 countries contributed to the discussion group; the vast majority of posts being from members in the United Kingdom. Activity within the group was consistently high for the first five years however, activity within the group declined in the final three years. Six purposes for which members use the online discussion group were identified: seeking and giving advice, networking, requesting and sharing material resources, service development, defining the role of occupational therapists, and student learning.

**Conclusions:**

Findings suggest that health professionals in specialized and often isolated areas of practice are keen to connect with colleagues and learn from each other’s experiences. The main purposes for which the online discussion group was used could be summarized as communication, information sharing and networking; though activity within the group declined significantly during the last three years of the data collection period. This raises questions about the sustainability of online discussion groups within the rapidly developing social media environment.

## Introduction

The process of globalization has rapidly increased over the last two decades and the flow of people, ideas, culture and technology is increasingly integrated. Internet based technologies are changing the way we communicate and disseminate important information. Online discussion groups, typically based on common interests, provide easy, real time access to information that were not readily available before the relatively recent technological and infrastructure improvements.

In 2003 an online discussion group called “forensic occupational therapy” [1] was established by one of the authors (ED) in the United Kingdom using the social media services of Yahoo! Inc. Forensic occupational therapy is understood as intervention for people with mental health problems who have committed a criminal offense, "acknowledging the important link between occupational behaviour and well-being" (p.13) [2]. The evidence-base for forensic occupational therapy is relatively weak [3] and forensic occupational therapists are often dispersed with limited numbers. The online discussion group was created following a national conference of approximately 100 forensic occupational therapists in the UK in late 2002. Some delegates at the conference shared their frustration of professional isolation, and their desire to continue the professional relationships and networking they had established during the conference. Whilst the discussion group was primarily formed to address this need, it was also recognized that such a group had the potential to develop an international network of forensic occupational therapists and could, through increased communication and networking, lead to the development of novel research collaborations to improve the profession’s evidence-base. Ten years later, the discussion group has an international membership of over 700.

The forensic occupational therapy group was examined as a case example of an online discussion group that brought together specialized clinicians, who otherwise found themselves quite isolated. Considering it is an emerging area of practice, membership of the group was strong and it exceeded initial expectations. Given the decline in activity of the group, questions have arisen about the life-span and potential of the social medium currently used. The objective of this study was to explore the purposes for which a disparate group of specialist practitioners used an online discussion group between 2003 and 2011.

## Background

Online discussion groups have been growing in size and popularity among health professionals. By 1998 there were over 85,000 online discussion groups available on a wide variety of health topics [4]. By 2004, a single critical care medicine discussion group had approximately 10,000 hits per year and over 1000 members including nurses, physicians, pharmacists, researchers and allied health care professionals across six continents [5]. Online discussion groups are used by health providers to build professional support networks and decrease isolation in smaller, less resourced areas [6], for professional development and keeping up to date on changes in an area of specialty [7], for achieving cultural competency, and for educating students [8].

Researchers studying the characteristics of online discussion groups found that group operators/moderators modeled appropriate communication and kept the discussions focused; members exchanged technical information and advice on how to communicate with health professionals [9]. Content analysis of a Spanish language online group revealed that postings covered scientific information (44%), discussion (22%), announcements (16%), noise or spam (13%), and clinical cases (5%) [10]. Whilst most questions posted were answered, it was suggested that having more active members and improved content quality would enable more ambitious online projects in the future [10]. Thomas & James [11] used author and content analysis along with a questionnaire survey to study one month’s postings for an online group of general practitioner physicians. Although considered an academic listserv, the group was perceived to be a support network; humour was used for 79% of the postings. While members were not required to submit posts, ‘lurkers’ – that is those people who read the list and do not contribute to discussion – were a growing proportion of the membership and were considered a problem, as they did not aid in the development of the group. Although this was a resource for the ‘lurking’ members, this resulted in a core group of practitioners ‘taking over,’ which was a concern for some members.

An American nursing organization created an online discussion group to provide opportunities for networking internationally and most members are from developed nations such as Australia, Canada, Finland, Ireland, Portugal, Slovenia and the United Kingdom, raising concerns about access for health professionals in developing nations [12]. Topics of discussion included professional and clinical issues, disability and impairment, administration and regulation, cultural and international issues, and housekeeping issues. It was concluded from the content analysis that the online group provided quick access to peers, an area to share information and a unique support system. Nurses identified the online group as a support network and an arena to keep up to date on nursing practice. The participants felt the entries provided credible information that they often shared with colleagues who were not members of the online group [13].

A preliminary scan of Internet based groups revealed at least 700 groups dedicated to occupational therapy and/or occupational therapists around the world. However, very little has been published on the purpose and usefulness of these groups. Occupational therapists in Atlantic Canada have explored the use of online discussion groups, or ‘on-line communities of practice’, to enhance communication between occupational therapists in similar areas of practice, to increase the speed of obtaining evidence for practice, and as a means of professional development for continuing competency requirements with their regulatory colleges [14]. These online groups brought together and supported occupational therapists who were geographically separated, and who did not have the time or resources to engage in research on their own [14].

As the Internet is a relatively new medium for obtaining information, there are not clear guidelines or standards of practice established for the health care community. This opens up a number of ethical considerations, such as reliability of information, privacy, confidentiality, and consent for research participation, when contributing to and extracting information from online discussion groups. The ideas and suggestions posted may not represent best practice and are not a substitute for the evidence available in peer-reviewed literature [15,16].

## Methods

The forensic occupational therapy online discussion group was selected as a single case example representing a point of convergence for occupational therapists who tend to be quite isolated from other occupational therapists in similar practice settings. There were no requirements or limitations on group membership and there was no expectation of privacy from the general public. Therefore, the data was accessed as publicly available information and ethics board review was not required in accordance with article 2.2(b) of the Tri-Council Policy Statement: Ethical Conduct for Research Involving Humans [17], which is the governing document for conducting ethical research in Canada.

### Statement

A case study method was used [18] to access and analyze eight years of online postings in the forensic occupational therapy discussion group beginning September 1, 2003 through August 31, 2011. The data was ‘cleaned’ , removing spam, administrative posts, repeat and blank posts. The remaining posts were coded to identify the purpose of each post and the topics being discussed. A theoretical thematic analysis [19] was then completed to describe general trends or patterns in the purposes for which members posted within the online discussion. The first author and four research assistants used a semantic approach to generating a list of codes for the entire data set. Portions of the data set were coded by more than one person for inter-rater consistency in thoroughness and differentiation throughout the coding. The first author sorted the different codes, refined the identified themes, and wrote a detailed summary of each theme. This analysis was then reviewed and further refined by the second author. Descriptive quantitative data were also collected including number of posts per year, number of posts coded within each theme, and the country where each post originated. Country of origin was identified either through statements within the post indicating where the occupational therapist is located or from the signature line of the author of the post.

The findings presented here provide a general understanding of the purposes for which the group was used as well as strengths and limitations of the current group structure in light of technological advances. Quotes from the data are presented in italics along with the date it was posted to the online discussion group.

## Results

### Demographic & usage characteristics

The discussion group grew steadily once it was established in 2003. The country where members were located could be identified for 67.5% of postings (n=1684). The vast majority of these postings were made by members located in the United Kingdom (88.2%, n=1485) with sporadic contributions from members in 20 countries around the globe (see Table 1).

**Table 1:** Country where each post originated

**Location**	**Postings**
UK	1485
Unknown	810
Australia	58
USA	32
Canada	26
Hong Kong	15
New Zealand	12
Cyprus	8
Japan	7
Singapore	7
Norway	6
Sri Lanka	6
Belgium	3
France	3
Germany	3
India	3
Ireland	3
Sweden	3
Czech Republic	1
Denmark	1
Malaysia	1
Taiwan	1
**TOTAL**	**2494**

Within the first year members posted 240 times. Posts increased by 42.7% to 419 in the second year and this rate of posting continued for four years (see Table 2). Consistently over time, December was the least active month within the group, while January and February were the most active. From September 2008, postings began to decline and continued to decline steadily through 2011. Over this three year period, the number of postings declined by 71%.

**Table 2:** Number of posts per year

**Year**	**Postings**
2003-04	240
2004-05	419
2005-06	359
2006-07	468
2007-08	441
2008-09	248
2009-10	191
2010-11	128
**TOTAL**	**2494**

### Online discussion group usage

Thematic analysis focused on the purposes for which members used the online ‘forensic occupational therapy’ group. Five clear purposes were identified (see Table 3): seeking and giving advice, networking, requesting and sharing material resources, defining the role of occupational therapists, and student learning.

**Table 3:** Frequency of each purpose for using the online discussion group

**Purpose**	**#(%) of posts**
Seeking and giving advice	1010 (40.5)
Networking	680 (27.3)
Requesting and sharing material resources	485 (19.4)
Defining the OT role	199 (8)
Student posts	129 (5.2)

#### Seeking and giving advice

Members used the online discussion group to seek and give advice on various aspects of practice; in particular in regard to specific interventions and service development.

### Specific interventions

Specific interventions were commonly discussed in the online group as the members asked for suggestions and examples from the experience of others. Members sought advice on the most effective means for carrying out specific interventions ranging from assessment to outcome measurements, as well as implementation of theoretical frameworks and models such as the Model of Human Occupation [20] (a profession specific model of practice) in forensic settings.

Information was requested about interventions and activities that may raise concerns in secure environments where patients have restricted access to certain items, – “*We are having clinical debates at present about the appropriateness of patients within medium secure services having MP3 players due to their recording capabilities*. *I guess this feeds into the greater considerations of technology*… *be great to hear what other services are doing*” ^(November 9, 2007)^.

Members were searching for information on various strategies including how to motivate clients to increase involvement in occupational therapy services – “*I have a good working relationship with these patients but find that they are very unmotivated to do any ward based activities as well as involving them in groups or individual* [*activities of daily living*] *sessions*… *has anyone got any ideas that they wish to share in terms of gaining interest in participating*?” ^(September 13, 2007),^ and how to work with people of various age groups –

“We are having a female unit built for the teenagers who are currently detained on a wing in the adult prison. My colleague… is keen to have a snoezelen room… I do not feel I have enough experience to advise regarding the use of snoezelen with teenage young offenders and would really be interested to hear from anyone who has used snoezelen within the psychiatric field or… how it could be used safely with this type of client group” ^(February 8, 2005)^.

#### Service development

As members planned new or improved programs, they requested information, advice, and material resources that will assist them in developing new services and/or revising existing ones. Information concerning best practice guidelines, needs assessments, program planning, and clinical protocols were frequently exchanged for this purpose. In particular, protocols and guidelines regarding non-smoking regulations, camera use, internet use, and how to adjust to changing governance mandates and structures were discussed – “*No one seems to be discussing the Agenda for Change* [*a UK classification*] *for* [*occupational therapists*]. *Is this because it is not an issue or is it that it is not happening to you*? *I would like to hear from anyone who has an opinion on the subject*, *particularly where they feel the different grades should be*/*will be* [*classified*]” ^(April 8, 2005)^. Members requested advice on a range of policy issues from current practice standards on the use of technology with clients, to ethical issues such as client possession of graphic material – “*have recently had problems with graphic material being found in the* [*regional secure unit*]. *Anybody have any ideas how to prevent this*??? *i*.*e*. *do you have an effective policy in place*?” ^(April 25, 2006)^.

Various assessments, interventions, and training programs were shared such as a variety of group interventions, animal therapy, clubs, and day programs – “*I am involved in what essentially is a creative writing group*… *called* ‘*words and images*’… *However we are finding that patients who suffer from hallucinations feel that this group is not appropriate for them*… *If anyone runs*, *or previously has been involved in creative writing I would be interested to hear about your experience*” ^(March 30, 2006)^. Workload measurement and documentation guidelines were also discussed as members sought more uniformity in these aspects of practice.

#### Requesting and sharing material resources

Common material resources such as journal articles, internet links, and assessment tools were sought and exchanged to provide evidence for best practices in areas such as specific interventions, occupational assessment, and application of theory – “*Does anyone have any info on or references about the Model of Creative Ability*? *I*’*m wondering if this would be usefully used in an intensive care situation and can*’*t find any stuff on it*” ^(February 10, 2006)^.

Members shared policies, protocols, and handbooks they had developed themselves – “*I have put together a* ‘*workbook*’ *that I have grandly named the* ‘*The Discharge Survival Guide*’. *I would be more than happy to share the information with you*, *if you*’*re interested*?” ^(September 12, 2006)^ as well as various facility employment protocols, policies, and intervention guidelines – “The unit is establishing a forensic community mental health team… I have been given the task of trying to locate information so can I ask is there anyone working in forensic community mental health teams who would be willing to share information such as job descriptions, policies and procedures?” ^(November 24, 2003)^.

#### Networking

The networking activities of the online group served three distinct purposes: communication about specific events, direct personal contact, and development of group dynamics.

The online group was used to communicate and coordinate local special interest group meetings within the United Kingdom – “*Please find attached the flier for the next South East Forensic* [*Occupational Therapy*] *Peer Support Group*. *It would be great to see you all there*. *Please try and make it if you can* – *this is a* free *event*!” ^(February 10, 2006)^. The group was also used to advertise national and international conferences in forensic occupational therapy and forensic mental health. Members often encouraged one another to attend and present at these meetings and conferences – “I have just posted a flyer on the group’s website for the forthcoming [International Association of Forensic Mental Health Services] conference in Edinburgh. The local conference organizing committee has agreed in principle to have a section meeting specifically for forensic occupational therapists… Abstracts will be submitted as usual to the conference. It is my intention that this meet could act as a focal point to develop active research networks and real research collaborations” ^(August 26, 2008)^.

There were a great number of requests for direct personal contact among group members. Individuals exchanged phone numbers, email addresses, and arranged for meetings and/or tours – “*You contacted me about prison work and possibly visiting our program*… *feel free to contact me on the above email*” ^(December 15, 2005)^ – as they looked for someone else who may have dealt with the same issues or concerns.

While most of the themes highlight a specific purpose for group members, this theme captured posts that served group process rather than content. These postings expressed personal greetings, appreciation, or apology – “*Thanks for the suggestions which are much appreciated*” ^(September 15, 2010)^. While these postings do not contribute to development of a particular topic, they contribute significantly to the development of group dynamics and inter-personal relationships between members.

### Defining the role of occupational therapists

The role of occupational therapists in forensic settings was often raised by students and new members posing questions to the veteran members of the group. Many aspects of the occupational therapist’s role sparked interesting debates such as the role of occupational therapy in diversion therapy or in application of restraints – “*The issue of* [*occupational therapists*] *doing control* &*restraint has been raised in our unit recently*. *There are mixed views amongst the team but for various reasons*, *possibly political*… *the decision has been made that* [*occupational therapists*] *will no longer participate in physical restraint*. *Does anyone have experience of arguing for* [*occupational therapists*] *doing control* &*restraint or have good reasons why we shouldn*’*t*?” ^(September 16, 2007)^. Ethical enquiries also sparked such debates, such as the role, or potential role, for occupational therapists in working with serial killers.

Client-centered therapy and the therapeutic relationship, their definitions and implications in forensic occupational therapy were examined, as well as who the intended clients should be (those with mental health conditions vs. general population in secure settings) for this area of practice –“*In forensic mental health the client is not necessarily the patient*. *Often the client is the* [*government department*] *or the court or the public*, *whose safety we are maintaining*. *This might explain why some of the* (*very clever*) *answers in other posts seem so complicated*” ^(November 7, 2006)^.

Practical and philosophical advice was exchanged about working from a client- centred approach and establishing service priorities – “*I am an* [*occupational therapist*] *working at a maximum security prison*, *drug and crime treatment*, *and early release ward*… *I wonder if there are others who work as I do and how they plan their work environments*, *are they alone as I am*, *do they counsel other faculty*, *manage a ward*, *implement activities or all of the above*?” ^(January 24, 2007)^.

Advice was exchanged about the role of occupational therapists in forensic settings including the occupational therapists’ role in risk assessments – “*Myself and a colleague are embarking on a research project and are aiming to focus on* [*occupational therapy*] *and risk assessment within a medium secure environment*. *This may focus on creating a pathway of assessment as we would a pathway of care to ensure a methodical approach is followed to enable accurate assessment of risk in different environments*. *If anyone has done anything similar or could help in any way please contact me*” ^(June 16, 2004)^.

Members also exchanged advice on working within a multidisciplinary team, including controversial areas of practice – “*If* [*occupational therapists*] *took a view that they shouldn*’*t get involved in* [*prevention and management of violence*] *it would be a retrospective step for the profession*. *Resources* – *or lack thereof*, *other commitments*, *other priorities are*, *in my view*, *not acceptable arguments to distance a* [*multi*-*disciplinary team*] *member from engaging in all aspects of forensic care*, *which on occasion will include managing violence and aggression*” ^(September 17, 2007)^. Members used the discussion group for an open dialogue to freely discuss their opinions on particularly sensitive questions regarding their role that are often not found in the literature.

### Student learning

Finally, the group was used for student learning. Students often posted questions related to fieldwork preparation, potential employment opportunities, and/or for assignments that were part of their course work – “*I am a 4th year* [*occupational therapy*] *student*… *I am writing a programme about sensory modulation techniques in a forensic psychiatric hospital*. *I saw that this topic was addressed a few years back on this list*. *I am wondering if you have anything more up to date to add to what is already in the archive*? *I*'*d particularly would like to know how successful you have been administering the sensory profile to your clients*, *how clients take to their sensory kits or the sensory room*?” ^(March 18, 2009)^. There was some debate within the online discussion about the appropriateness of student postings however, the general consensus among members was that questions from students prompted reflective responses from experienced occupational therapists on topics they might not otherwise have actively considered or discussed.

## Discussion

This study investigated the use of the forensic occupational therapy on-line discussion group [1], which used Yahoo Groups as its platform, as a case study for the use of social media for healthcare professionals who work in small or isolated groups. The major functions for which this group has been used over the 8 year period can be summarized as:

Communication and information exchange between specialist practitioners, researchers, and students.

Coordinating networking events at local, national and international levels.

It is clear from the data that, while membership and involvement in the group has spread throughout the world, the group continues to be dominated by members in the United Kingdom and postings reflected issues and concerns relevant to practice in that country. This is not surprising considering that the group originated there and forensic occupational therapy features prominently within the broader occupational therapy community in the United Kingdom. Therefore, patterns in usage may continue to be shaped by the United Kingdom experience and may not reflect priorities for members in other countries, particularly those in developing and/or non-English speaking countries. Over time membership spread throughout the world however security protocols inherent within the online platform limit the potential for identifying where in the world members are located, preventing an accurate geographical description of group membership.

Online discussion groups connect individuals with a common interest across diverse geographical regions. Such groups are increasingly popular among health professionals who are faced with heavy workloads, competing priorities, and limited resources for professional development. Social networking theories are emerging that describe learning in the digital age as a prevalently social activity [21-23]. These theories, particularly the theory of connectivism [22], suggest that knowing and learning are defined by connections and that learning has become primarily a network forming process [21]. The extensive membership (>700) of the forensic occupational therapy online discussion group suggests that health professionals in specialized areas of practice are keen to connect with colleagues and learn from each other’s experiences. The group allows individuals who practice in small groups or are in isolated areas of practice to have a common meeting place to exchange information, ideas and resources. This allows for increased access to experiential evidence in a field where empirical evidence is limited and challenging to locate.

However, the decline in activity over the last three years raises concerns about the sustainability of this and potentially other similar groups. The reasons for this decline are unknown. Potential causes could include a change in leadership within the membership, or developments in newer social media platforms, such as Facebook and Twitter, which pose a challenge for professional networking globally. The primitive functioning of the web-based discussion group form of social media also illustrates its limitations in functionality; with key features, such as a search facility, that are now common place in other social media platforms absent from the study discussion group. Reviewing the postings during analysis it was evident that when new members joined the group, topics central to forensic occupational therapy were repeatedly discussed. Newer forms of social media have clear functional advantages that would enable members to search previous posts more efficiently.

Whilst the forensic discussion group appears to have been used extensively for communication, information sharing and networking, certain activities that could have been foreseen are noticeable by their absence. Specifically, considering the relative weakness of the evidence base for forensic occupational therapy, this group had potential to be used to initiate collaborative research activities or for disseminating research findings. This does not appear to have occurred. The reasons for this cannot be drawn out of the current study, however it suggests that whilst social media platforms do lessen professional isolation and increase communication, this alone is insufficient to change certain behaviours, such as the lack of collaborative research activity within forensic occupational therapy [24].

### Limitations and recommendations

Theoretical thematic analysis is an analyst-driven process that involves coding data for a specific purpose; in this instance understanding the purpose of one online discussion group for forensic occupational therapists. Consequently the study provided a more detailed analysis of the purposes for which members were using the online discussion group, but a less detailed analysis of the data overall. A deeper, interpretive analysis of the topics discussed by the group is now being undertaken. This was not within the scope of this study; however, it is now apparent that such a study is valuable as it will further enhance understanding of the purposes for which members used the online discussion group.

Despite the declining use of this specific group, the structure and accessibility of social media in general has been demonstrated through the forensic occupational therapy discussion group, as well as amongst [5-11]. The results indicated the ability of social media to increase networking, decrease professional isolation and encourage the sharing of ideas among group members. However, in an era where occupational therapists are being increasingly trained as clinician-researchers, online discussion groups that engage professionals in specific areas of practice appear to be an ideal platform for the development of collaborative research activities and dissemination of research findings. To date, however, there is little evidence of this occurring within the forensic occupational therapy discussion group. While improved communication between members is important to the development of the profession’s research base, further research is required to investigate this issue in greater depth.

With the decline in use of the forensic occupational therapy online discussion group and the ever-changing social media technologies, it would be prudent to investigate the potential future of this and other similar groups. Key questions should cover issues such as:-Is the topic area still of interest? Do advances in social media offer alternative technologies or platforms that are better suited to the groups needs?

## Conclusions

This case study of an international electronic discussion group for forensic occupational therapists illustrates the value of social media as a platform to increase communication, information exchange and networking between healthcare professionals with a shared interest and who may be isolated within their practice area. The international nature of the membership also illustrates the advantages that such a resource has in sharing and developing practice between nations. As the group was developed on an early web-based discussion group platform it is does not have the functionality that newer and more popular social media sites have. This limits the abilities of the current site and may be contributing to the noticeable decrease in its usage. Consideration of sustainability and ‘future proofing’ should be given when developing any new social media platform resource for health professionals, as migrating members from one platform to another is a significant task and likely to result in loss of members in the process.

## Competing interests

Both authors declare that they do not have any financial or non-financial competing interests in relation to this paper.

## Authors’ contributions

CD extracted the publically available data, carried out initial coding analysis and secondary thematic analysis, and drafted the manuscript; EASD conceived of the study, participated in its design, and helped draft the manuscript; all authors read and approved of the final manuscript.

## Authors’ information

CD, PhD, OT Reg(NS), is an Assistant Professor in the School of Occupational Therapy at Dalhousie University in Halifax, Canada. Her research is focused in the areas of forensic occupational therapy, the criminalization of people with mental health problems, and the social organization of mental health care within criminal justice systems, EASD, BSc(Hons), Dip. CBT, PhD, is a senior research fellow at the Nursing Midwifery and Allied Health Professions Research Unit, University of Stirling, Stirling. His research focuses on investigating, developing and evaluating the quality and practice of direct patient care. He is the founder and moderator of the Forensic Occupational Therapy discussion group that is investigated in this paper.

## Pre-publication history

The pre-publication history for this paper can be accessed here:

http://www.biomedcentral.com/1472-6963/13/253/prepub
